# The Isolation of Nucleic Acids from Fixed, Paraffin-Embedded Tissues–Which Methods Are Useful When?

**DOI:** 10.1371/journal.pone.0000537

**Published:** 2007-06-20

**Authors:** M. Thomas P. Gilbert, Tamara Haselkorn, Michael Bunce, Juan J. Sanchez, Sebastian B. Lucas, Laurence D. Jewell, Eric Van Marck, Michael Worobey

**Affiliations:** 1 Department of Ecology and Evolutionary Biology, University of Arizona, Tucson, Arizona, United States of America; 2 Ancient DNA Laboratory, School of Biological Sciences and Biotechnology, Murdoch University, Perth, Western Australia, Australia; 3 National Institute of Toxicology and Forensic Science, Canary Islands Delegation, Tenerife, Spain; 4 Department of Pathology, St Thomas' Hospital, London, United Kingdom; 5 Department of Laboratory Medicine and Pathology, University of Alberta Hospital, Edmonton, Canada; 6 Department of Pathology, University Hospital, University of Antwerp, Antwerp, Belgium; Ecole Normale Supérieure de Lyon, France

## Abstract

Museums and pathology collections around the world represent an archive of genetic material to study populations and diseases. For preservation purposes, a large portion of these collections has been fixed in formalin-containing solutions, a treatment that results in cross-linking of biomolecules. Cross-linking not only complicates isolation of nucleic acid but also introduces polymerase “blocks” during PCR. A wide variety of methods exists for the recovery of DNA and RNA from archival tissues, and although a number of previous studies have qualitatively compared the relative merits of the different techniques, very few have undertaken wide scale quantitative comparisons. To help address this issue, we have undertaken a study that investigates the quality of nucleic acids recovered from a test panel of fixed specimens that have been manipulated following a number of the published protocols. These include methods of pre-treating the samples prior to extraction, extraction and nucleic acid purification methods themselves, and a post-extraction enzymatic repair technique. We find that although many of the published methods have distinct positive effects on some characteristics of the nucleic acids, the benefits often come at a cost. In addition, a number of the previously published techniques appear to have no effect at all. Our findings recommend that the extraction methodology adopted should be chosen carefully. Here we provide a quick reference table that can be used to determine appropriate protocols for particular aims.

## Introduction

The recovery of nucleic acids (DNA and RNA) from fixed, paraffin-embedded specimens is challenging. Although formaldehyde (HCHO), a principal ingredient of most commonly used fixatives, does not physically degrade nucleic acids *per se*, it leads to the generation of DNA-protein [1]–[6] and RNA-protein [7] cross-linkages. Furthermore, the nucleic acids will fragment in situations where the fixative solution is unbuffered, as the pH can be extremely low (<1). Both the above provide serious challenges to genetic studies developed around the polymerase chain reaction (PCR) through a reduction in both the amplifiable quantity, and length, of DNA/RNA.

Despite these problems, however, a large number of fixed specimens are held in collections across the world, and the human and pathogen genetic information they contain is often critical to important health-related investigations. Therefore, the development of any methods that aid the recovery of optimal quality nucleic acids is desirable, and indeed a large number of papers have previously been published on these matters. However, despite their quantity, few if any have provided directly comparable results as to the relative efficiency of the many described methods. This is due to a number of factors. Firstly, many different measures of nucleic acid quality exist, and they are not always comparable. Secondly, the nucleic acid quality within specific fixed specimens is highly dependent on a large number of parameters that can lead to the degradation of nucleic acids. These include pre-fixation factors (e.g. tissue type and amount, degree of autolysis); fixation related factors (e.g. pH, temperature, and duration of fixation, as well as which fixative was chosen); and post-fixation factors (e.g. temperature and duration of storage) [8]–[11]. As such, unless identical data sets and measures of nucleic acid quality are used between trials, it is very difficult to draw any meaningful conclusions. This may in part explain why it is not unusual to find conflicting findings in previously published studies. For example, in a comparison of the effect of time of incubation during tissue digestion, Isola et al. [12] argue that prolonged time is better, while Banerjee et al. [13] argue that no more than 3 hours are required.

In this paper we report the results of a comparison of a large number of published methods that deal either with the recovery of nucleic acids from fixed specimens, or their subsequent manipulation to increase their quality. The aim of this study was to generate data that can be used to help choose which method to apply under different endpoint requirements. To ensure that the results are maximally informative to allow retrospective genetic analyses, we have used a consistent set of historical specimens for all extraction methods, and assessed the quality of the nucleic acids using up to 10 different assays. The specimens themselves are all archival, ranging from 49 to 11 years in age, and have been fixed with either buffered or unbuffered formalin, or with Bouin's solution. Bouin's is a picric acid and formaldehyde containing solution that has historically been used predominantly in some European and European colonial laboratories [14] and is notable for its extremely low pH (<1).

Different characteristics of extracted nucleic acids may be viewed as important in studies with different aims (for example, some may require increased length of amplicon, while others may require increased effective amplifiable copy number, while others may simply require increased levels of extracted nucleic acids). Furthermore, because the above may not be linked in a straightforward manner, we have investigated the outcome of the investigated extraction methods using a range of quantitative and qualitative measures. This includes raw quantity of extracted DNA and RNA (independently measured), PCR amplifiable quantity of human nuclear and mitochondrial DNA (nuDNA and mtDNA), maximum amplifiable length of human nuDNA PCR product, effectiveness in human nuDNA multiplex PCR assays, amplifiable quantity of human RNA following cDNA synthesis through RT-PCR (reverse-transcription PCR), and effectiveness for both proviral DNA, and RT-PCR assays of viral pathogen RNA. Full details are described in the methods section. Because of this range of parameters, and although not a strictly accurate description, we henceforth refer to any increases in any of the chosen measures of nucleic acids as ‘increased quality’ of the nucleic acids.

## Materials and Methods

### Experimental Parameters Tested

Before detailing the particulars of the materials and methods we employed, in this section we aim to provide an overview of the many manipulations that can be used before, during, and after DNA/RNA extraction from fixed specimens. While many published studies contain techniques that are significantly different to those previously used, others are small modifications of previous methods. To test all variations would be well beyond the scope of any reasonable investigation. Therefore, we have isolated what we believe to be the key pre-, during-, and post-extraction protocols, so that their particular effects can be tested.

#### 1. Pre-extraction treatments of fixed tissues

Many studies apply pretreatment techniques prior to the extraction of the nucleic acids from the fixed tissues. A number of these are directly related to the removal of the paraffin wax in which many samples are stored. (Paraffin has commonly been used because it renders samples convenient for storage, and is a requisite of many microscopy-based analyses). The most common technique for paraffin removal is based on that described by Goelz et al. [15], using a progression of xylene (or other similar solvents) and ethanol washes (e.g. [16]–[19]). Alternative methods also exist, such as its removal through direct melting using microwaves [13]. The removal of paraffin is believed to be important–for example Stanta et al. [20] argue it otherwise leads to PCR inhibition during subsequent PCR. However, despite this, a growing number of authors take no specific steps to remove the paraffin [21]–[24] either due to its substitution with other steps, or simply due to a belief that its removal is unnecessary.

The removal of paraffin is not the only pretreatment method that has been described. Formaldehyde induces the cross-linking of protein to other molecules (including nucleic acids) in a manner that is to some extent heat reversible [6], [25]–[27]. As such, several pre-digestion heat treatments have been described that confer apparent benefits (with regard to DNA quality). These include a 15 minute pre-incubation of the fixed tissues at 98°C in a conventional Tris-based digestion solution (minus proteinase k which would be denatured) [21], or, at a greater extreme, pre-incubation at even higher temperatures (up to 120°C in an autoclave) in alkali solutions [22], [23].

A further pretreatment that has been reported with specific regard to tissues fixed using Bouin's solution is washing (after paraffin removal) in 27 mM LiCO_3_ solution [17]. Although this has not been used in other studies on Bouin's-fixed samples (e.g. [14]), and in the original paper the authors do not justify why this is necessary, we presume the aim of the wash is to help remove picric acid from the tissue slices, which may have a detrimental effect, at later stages, on the nucleic acids. From our own observations, we note that the characteristically bright-yellow Bouin's-fixed tissues rapidly lose their yellow color after one to two brief (30 second) incubations in the LiCO_3_ solution. However, it remains unclear whether this step is actually warranted to enable the recovery of higher quality nucleic acids.

One of the more interesting pretreatment methods that has been described is the application of graded ethanol washes (for example a series of 30%–100% ethanol washes in 10% increments) followed by critical point drying aimed at removing formaldehyde from specimens [28]. Critical point drying is a technique usually associated with the preparation of samples for high-resolution microscopy through the use of liquid carbon dioxide, pressure, and temperature, to rapidly desiccate the specimen. Although Fang et al. [28] do not provide a clear explanation as to why this should efficiently remove formalin from fixed specimens, their results indicate increases in both DNA yield, and maximum size of PCR amplifiable fragment, from 403 to 1844 base pairs (bp), following the application of the technique. In comparison to the success of other published studies, the reported success is remarkable. However, from a practical viewpoint, there appears to be no logical explanation as to why this method works; furthermore, we note that there has been a lack of subsequent published interest in this method.

#### 2. Nucleic acid extraction methods

Following any pretreatment that may have been applied, a number of methodological variants have been published for subsequent nucleic acid recovery and purification. While some differ because they are designed to specifically target DNA or RNA, others are described as designed specifically with the fixation chemistry in mind.

Although some studies have used Chelex-based extraction methods, these have been demonstrated to be inferior in several other studies (e.g. [29], [30]). Therefore, we have not investigated their use further. One alternative that has been used in a number of previous studies is the digestion of tissues using what we henceforth term ‘regular’ digestion buffers. Specifically, this refers to the wide range of related digestion buffers that are composed principally of Tris-HCl, EDTA, NaCl, detergent and proteinase k (e.g. [16], [17]). Some studies have developed this buffer further, through the addition of binding agents aimed at the removal of formaldehyde that might be released during tissue digestion. For example, Shedlock et al. [31] recommend the inclusion of glycine for this purpose. Following incubation in such buffers, nucleic acids are usually purified using organic extraction methods such as sequential phenol:phenol:chloroform extraction (e.g. [16], [32]), and then either directly used for subsequent PCR or cDNA synthesis, or further concentrated through precipitation of the nucleic acids using ethanol or isopropanol (e.g. [16]).

The predominant alternative to Tris-based digestion buffers are guanidinium thiocyanate/proteinase k containing buffers, favored by those who perform DNA extractions using commercially available kits (e.g. Qiagen's QIAamp DNA micro kit) that are based on the silica-binding principle described by Boom et al. [33] (e.g. [18], [21], [32], [33]).

Although most studies incorporate proteinase k digestion as described in the methods above, recently several studies have been published that indicate this may be unnecessary. These include the studies of Shi et al. [22], [23] that report the recovery of increased quality DNA following short incubation in hot alkali (80°C–120°C), directly followed by organic purification.

The composition of the extraction buffer is not the only potentially variable parameter with regard to nucleic acid extraction. In light of early observations that cross-linking of DNA is at least to some degree heat-reversible [6], [25], [26] it is logical that increased exposure to thermal energy, whether through increasing incubation temperature [22] or increasing incubation time [12], may be beneficial. However, as nucleic acid degradation is also clearly linked to temperature (e.g. [34]), such steps ultimately will involve a trade-off between heat-induced nucleic acid degradation and cross-link reversal.

#### 3. Post-extraction manipulation of DNA

One of the more interesting recent developments with regard to genetic analyses on fixed specimens is the possibility of damage reversal through the use of *Taq* DNA polymerase as a means to repair nicked single-stranded DNA [14], [35]. The authors have demonstrated its use on both formalin and Bouin's-fixed specimens, although not in combination with other methods designed to reverse cross-linkages.

### Materials

All experimental tests were performed on microtome sections of fixed, paraffin-embedded, archival human tissue. Rigorous attention was given to preventing cross-contamination between samples by using fresh blades for each specimen and cleaning the microtome with ethanol and bleach solutions between specimens. The experimental design was such that tests were always performed on paired samples, with one sample as a control and one being manipulated. This pairing ensured that in all tests comparisons of the effect of the various techniques could be made using near identical tissue samples. Although all paired samples were sourced from the same paraffin embedded tissue blocks, naturally small variations in the tissue within the block will lead to small variation within the pairs. However the incorporation of multiple pairs of tissue for each test helped ensure that the effects of such intra-sample variation were kept to a minimum.

In total 180 extractions were performed, sourced from 11 different paraffin embedded tissues sampled between 1958 and 1996. Four of these had been fixed in Bouin's solution, while the remaining seven had been fixed in formalin (either unbuffered or buffered). These latter seven were sampled from HIV-1 infected individuals. For full details of the sample sources see Table 1. Between 3 and 10 microtome slices (from 5–10 µm in thickness, consistent width within pairs) were used per sample. Final amounts of tissue within paired samples, measured via post-deparraffinisation mass (unless paraffin was specifically not removed) were always very similar–within 10% of each other. The pairs of tissue slices were then subjected to one of the range of as detailed below. Following extraction the nucleic acid quantity and quality were assayed from each using a range of tests as detailed below.

**Table 1:** Details of samples used in this study.

Sample ID	Tissue	Fixative	Age	Origin	HIV-1	Source	Extractions
783	Kaposi's Sarcoma	Bouin's solution	1960	DRC	No	Van Marck	7
829	Kaposi's Sarcoma	Bouin's solution	1958	DRC	No	Van Marck	7
1029	Lymphoma	Bouin's solution	1960	DRC	No	Van Marck	7
1536	Lymphoma	Bouin's solution	1959	Rwanda	No	Van Marck	14
A01	Lung	Bouin's solution	1981	Belgium	Yes	Van Marck	3
PM80	Lung	buffered formalin	1980	Canada	Yes	Jewell	8
PM78	Spleen	unbuffered formalin	1991	Cote d'Ivoire	Yes	Lucas	23
PM82	Spleen	unbuffered formalin	1991	Cote d'Ivoire	Yes	Lucas	21
PM85	Spleen	unbuffered formalin	1991	Cote d'Ivoire	Yes	Lucas	38
PM88	Spleen	unbuffered formalin	1991	Cote d'Ivoire	Yes	Lucas	7
PM96	Lung	unbuffered formalin	1996	Canada	Yes	Jewell	45
							180

### Methods

#### Comment on experimental approach adopted

As detailed above, a number of variables exist that may influence the quality and quantity of nucleic acids recovered from fixed specimens. As the aim of our study is to examine as many different variants as possible, in order to provide preliminary data as to their relative performance, we have designed our experimental approach to incorporate as many variables as possible, on a consistent panel of specimens. Using treatment/control pairs from this panel, we aim to produce data that allow for the efficiency of the methods to be meaningfully compared. We acknowledge the inherent trade-off in statistical power compared to studies focusing on larger numbers of specimens but fewer methods; nevertheless, we feel that the results of our investigation will be especially useful due to their breadth and due to the advantages of comparing carefully controlled treatment/control pairs. The incorporation of paired specimens for every variable we tested enabled us to dissect the data set into the individual treatments during the data analysis. However, as a result of both this, plus analytical limitations, the final number of paired samples examined for each treatment, and each analysis within treatment varied significantly. Full details of how many samples were investigated for each permutation are detailed in supplemental table S1.

#### Pre-extraction treatments: the effect of deparaffinisation

The effect of deparaffinisation was performed through the comparison of treated samples against untreated controls. Paraffin was removed predominantly through immersion in 100% xylene following Krafft et al. [16]. Briefly, samples were immersed in xylene for 5 minutes, centrifuged to pellet the tissue and, to enable removal of the xylene, washed twice in ethanol (1×85% and 1×100%), then allowed to air dry at 75°C for 5 minutes. For a number of samples that were used in later tests, the xylene was replaced with 100% pentane, which is more volatile than xylene, thus much easier to remove from the sample. Although not specifically tested in this study, we saw no evidence in preliminary tests that the choice of solvent affected results.

#### Pre-extraction treatments: the effect of 27 mM LiCO_3_ on nucleic acids extracted from Bouin's-fixed tissues

The potentially beneficial effect of LiCO_3_ on Bouin's-fixed tissues was tested through immersion of deparaffinised tissues in 1 mL 27 mM LiCO_3_ for 3–5 minutes (following [17]). Following removal of the LiCO_3_ extractions proceeded as normal.

#### Pre-extraction treatments: the effect of 98°C heat pretreatment

The effect of a heat pretreatment step prior to nucleic acid digestion was investigated, as described by Wu et al. [21]. Specifically, relevant digestion buffers were added to tissue samples without the addition of proteinase k, and subsequently heated for 15 minutes at 98°C. Immediately after, the samples were cooled on ice, and proteinase k was added as required in the relevant digestion buffers. Subsequent extractions continued as normal.

#### Extraction treatments: the effect of glycine in the digestion buffer

The addition of glycine during extractions from formalin-fixed tissues has been advocated by several authors (e.g. [31]) as a binding agent for released formalin. To test for any effect, pairs of samples were investigated, through digestion in either a ‘regular’ digestion buffer (25 mM Tris-HCl pH 8, 25 mM Sodium citrate, DTT, 2% SDS, 5 mM CaCl_2_, 2.5 mM EDTA, 50 µl 20 mg/ml proteinase k) or in a ‘glycine’ buffer, containing in addition 25 mM glycine. Samples were incubated with agitation at 55°C overnight. After incubation the samples were purified twice with phenol and once with chloroform [36], after which the nucleic acids were precipitated with isopropanol and glycogen following Krafft et al. [16]. The final nucleic acid pellets were resuspended in 100 µl TE, and stored frozen in 10 µl aliquots at −80°C until required for analysis.

#### Extraction treatments: the effect of ‘silica’ versus ‘organic’ nucleic acid purification techniques

The effect of silica versus organic extractions was assayed. Silica extractions were performed using QIAamp DNA micro extraction kits (Qiagen) following the manufacturer's guidelines, although using double the suggested volumes of all reagents prior to the wash stages (proteinase k, buffers AL and ATL, 100% Ethanol). DNA was eluted in 100 µl TE to be consistent with other extractions. Organic extractions were undertaken as described for the glycine-free buffer detailed above. The digestion times and temperatures varied by extract, in accordance with various tests described below; however times and temperatures were always consistent within pairs of samples.

#### Extraction treatments: the effect of incubation time

To investigate the effect of incubation time on nucleic acids, pairs of samples were tested across the following incubation times: 1, 6, 12, 24, 48, 72 and 96 hours.

#### Extraction treatments: the effect of incubation temperature

To investigate the effect of incubation temperature on nucleic acid quality, samples were compared across the following digestion temperatures: 55, 65, 75 and 85°C, using the QIAamp DNA Micro extraction kit (Qiagen).

#### Post-extraction treatments: the effect of Taq-based DNA repair

Bonin et al. [14], [35] have recommended the incubation of extracted DNA with *Taq* polymerase as a means to increase the amplifiable length of fragment. Although clearly effective under their extraction conditions, we investigated whether its effects may be negated by improvements conferred by any of the above techniques. Because this treatment is applied to the DNA extract and is not part of the extraction itself, in contrast to the other methods tested this was not performed on paired extracts. Instead, the comparison was made on treated and untreated aliquots from the individual DNA extracts themselves, using the method as described by Bonin et al. [35].

#### Additional investigations: effect of critical point drying and hot-alkali treatment

In addition to the above variables, we also performed some tests of the effect of two previously published protocols: the pretreatment of tissues using critical point drying [28], and the incubation of tissues in strong, hot alkali solutions [22], [23]. In comparison to the above investigations, these tests were limited due to resources and sample tissue. However, although the results generated might not be statistically significant, they still provide some evidence for or against claims of these previous studies.

#### Critical point drying

Critical point drying was performed on 5 subsamples from each of two fixed tissue specimens, PM78 and PM82. Each of the subsamples was deparaffinised as detailed above, then subjected to one of the following treatments.

i) Graded ethanol dehydration using 30,40,50,60,70,80,90 and 100% ethanol washes, followed by 100% isoamyl acetate wash, followed by critical point drying.
ii) Graded ethanol dehydration using 70,80,90 and 100% ethanol washes followed by 100% isoamyl acetate wash, followed by critical point drying
iii) 100% ethanol wash followed by 100% isoamyl acetate wash, followed by critical point drying
iv) Seven sequential 70% ethanol washes, followed by a single 100% ethanol wash followed by 100% isoamyl acetate wash, followed by critical point drying
v) As (iii) but omitting the isoamyl acetate wash.

Treatments (i–iii) were as described by Fang et al. [28]. Treatments (iv and v) were instigated by us in order to investigate whether any effect of treatment (i) was simply a result of seven sequential washes (in contrast to seven graded ethanol washes) (treatment iv), and whether isoamyl acetate was a necessary addition (treatment v).

#### Hot-alkali treatment

Shi et al. [22], [23] have published several variations on a hot-alkali based method that they report increases the quality of the DNA extracted from formalin-fixed specimens. We have partially repeated their experiments on a small number of samples, and incorporated several variations in order to further investigate their methods and to compare them with the other manipulations we considered.

Initially, tissue samples from three original samples were chosen and subjected to variations of Shi et al's original method [22]. Two of the samples were formalin fixed (samples PM85, PM96) while the third was fixed in Bouin's solution (sample 1536), a fixative not investigated in the original publication. The samples were subjected to 6 treatment regimes adapted from the original publication–incubation at two temperature regimes (100°C and 120°C for 25 minutes) in digestion buffer adjusted to three different pH values using NaOH (ph 7.8, 9.2 and 11). The digestion buffer was as described above, although with the omission of proteinase k. Following treatment, nucleic acids were extracted following the organic/precipitation method described above. The nucleic acids were assessed for both PCR amplifiable nuDNA and total DNA yields (as in the original paper), but also for PCR amplifiable and total RNA yields.

In addition, we also investigated Shi et al's updated method [23], in comparison to some modifications of our own. Subsamples from two formalin fixed samples (PM88 and PM96) were treated in the following three ways:

- 25 minute incubation at 120°C in 0.1M NaOH/1%SDS solution (pH 12.8) [23]
- 25 minute incubation at 120°C in a ‘regular’ digestion buffer (no proteinase k) as used above, adjusted to pH 11.2 using NaOH.
- As (b), although followed by an additional 24 hour incubation at 55°C with proteinase k

A third fixed sample (PM85) was subjected to the same three treatments, plus an additional three: the digestion for 48, 72 and 96 hours respectively at 55°C in the ‘regular’ digestion buffer (treatments d–f, respectively). This additional comparison enabled the efficacy of the hot alkali methods to be contrasted with the effect of simply elongating digestion times. The extracted nucleic acids from these additional experiments were quality assessed as in the original test.

### Nucleic acid quality assays

Due to both practical and resource limitations and due to observations arising during the data generation, not all methods tested were assayed with all nucleic acid quality indices. Full details of the indices used for each method are detailed in supplemental table S1.

### DNA assays

#### Total DNA yield

The absolute yield of extracted DNA was quantified using a NanoDrop ND-1000 (NanoDrop Technologies). With the exception of the samples that contained no DNA at all, the mean and median values of the DNA extracts were 169 and 54 ng/µl respectively. This is well above the minimum sensitivity of the Nanodrop ND-1000 (2 ng/µl). Therefore, we believe the measurements to be accurate reflections of the DNA concentrations within the extracts (within the published measurement error of ±2 ng/µl). Following initial quantification, all measurements were standardized to account for the final volumes of DNA solution (ng/µl) (this varied between extracts). Statistical analyses were performed on the absolute difference between DNA yields between paired specimens.

#### PCR amplifiable levels of nuDNA and mtDNA quality

The relative amount of PCR-amplifiable nuDNA and mtDNA were assayed in each pair or extractions using SYBR-green based quantitative real-time PCR assays (qPCR). The assays were designed to amplify a short fragment of nuclear and mitochondrial DNA, respectively. Primers Amelo2F (5′-CCCTGGGCTCTGTAAAGAATAGTG) and Amelo2R (5′-ATCAGAGCTTAAACTGGGAAGCTG) (24) amplify a 106/112 bp (Y chromosome and X chromosome, respectively) fragment of the single copy nuclear Amelogenin gene. Primers mtDNA16304F (5′-AACAAACCTACCCACCCTTAACAGT) and mtDNA16316R (5′-TGTGCTATGTACGGTAAATGGCTTT) amplify a 61bp fragment of the mtDNA Hypervariable Region between Cambridge Reference Sequence nucleotide positions 16280 and 16340 inclusive [37]. Dissociation curve analyses during initial optimization (and all analytical) qPCR assays indicate that these primer sets do not produce any secondary non-specific double stranded DNA products; thus, subsequently measured SYBR-green fluorescence directly represents the number of amplified Amelogenin and mtDNA fragments. It should be noted that although the size difference of the two targets makes comparison between the nuDNA and mtDNA yields meaningless, this is not the aim of the study and the qPCR data was used solely to measure the independent changes in nuDNA and mtDNA quality.

Real time PCR analyses were performed in 25 µl reactions, using an ABI PRISM 7700 thermocycler (Applied Biosystems, Foster City, USA) and SYBR-Green PCR Master Mix (Applied Biosystems), following the manufacturer's instructions. Each 40 cycle qPCR reaction was performed using 1 µl of DNA taken from a dilution series of at least 4 data points on each extract, in order to monitor for non-linear amplification behavior that may be attributed to PCR inhibitors present in the DNA extract or differences in qPCR efficiency. Using this information, cycle-threshold (Ct) values used in the analyses were accepted only on data generated in the absence of PCR inhibitors, and where the data indicated comparable qPCR amplification kinetics between different extracts for each primer pair. The Ct values were normalized against a panel of control extracts used in every independent reaction to provide directly comparable results between all extracts, representing a comparable Ct value per µl extract.

Final results were measured as Ct value. The reader is reminded that, firstly, in qPCR assays, lower Ct values indicate higher levels of PCR amplifiable template. Secondly, Ct values used in qPCR assays are recorded during the exponential phase of the reaction, and a 1 cycle difference in Ct value can be very roughly approximated to a 2 fold difference in levels of starting template. (If amplification efficiency is <100% then the true increase is <2 fold per cycle; given our aims, the exact relationship is not critically important). Statistical analyses were performed on the absolute difference of Ct values between paired specimens.

#### Size of PCR-amplifiable fragment

The size of amplifiable PCR product was assessed through a size assay on each extract, in order to investigate improvements in size of DNA fragment in each DNA extract. The assay was performed following Gilbert et al. [24]. In brief, each extract was subjected to two PCRs, targeting different nuDNA fragments within the single copy Amelogenin gene:

1. Amelo2F/Amelo2R (106/112bp sex-dependent, details as above);
2. Amelo1F/Amelo1R (212/218 sex-dependent 5′-ACCTCATCCTGGGCACCCTGG/5′-AGGCTTGAGGCCAACCATCAG) (38).

Twenty-five µl PCR reactions were cycled 40 times at 94°C for 30 seconds, 60°C for 1 minute, 72°C for 30 seconds, final extension 72°C for 7 minutes, and incorporated 1 µl extract, 0.1 µl Platinum Taq High Fidelity (5 U/µl, Invitrogen), 0.1 µl 25 mM dNTPs, and 300 nM each primer; the products were visualized by agarose gel electrophoresis. The primers have all been demonstrated, through previous sequencing analyses within our laboratories and elsewhere, to be specific to the designated targets. For analytical reasons, results were recorded for each extraction as maximum amplifiable size. For statistical analyses, categorical variables were assigned to the pairs of data to indicate either no difference, or a difference between paired samples for each of the two tests described above.

#### nuDNA quality via multiplex PCR with minisequencing

The quality of multiplex PCR amplifiable DNA was assayed using a recently published Multiplex PCR with Minisequencing (MPMS) assay. This system, modified from Sanchez et al. [39] simultaneously co-amplifies 44 autosomal unlinked SNPs in a single PCR reaction, using primers that amplify PCR products of between 19 and 115 bp in length. MPMS conditions were as detailed in Gilbert et al. [24] and the SNPs were genotyped using minisequencing. This assay has recently been validated on formalin-fixed paraffin embedded tissue [24]. In that study the authors demonstrated that the efficiency of the MPMS, as measured by percentage of the SNPs that amplify, correlates to some degree with the DNA quality of the extract, as measured both via quantitative PCR and maximum amplifiable size of PCR product. Final results were scored as number (out of maximum 44) of successfully minisequenced SNPs and statistical analyses were performed on the absolute difference in success between pairs.

#### Quality of proviral DNA

Seven of the fixed tissues investigated in this study came from individuals infected with HIV-1 (Table 1). Within an infected host, HIV-1 proviral copy number is typically much lower than even single-copy nuclear genes; HIV-1 DNA is thus harder to PCR-amplify. These samples therefore provide a further potential measure of DNA quality. HIV-1 proviral DNA was amplified using a set of primers that target a short region of the HIV-1 genome, and which have been demonstrated in other studies to be effective across all of HIV-1 group M [40]. Primers HIVG1 and HIVG2 amplify a 106 bp fragment of the *gag* gene [40]. PCRs were performed using the Amplitaq Gold enzyme system (Applied Biosystems, Forster City, CA). Each 40 µl reactions contained 2.5 pmoles each primer, 0.1 mM mixed dNTPs, 3.5 mM MgCl_2_, 4 µl 10×buffer, 20 µg BSA and 0.2 µl Amplitaq Gold (5 U/µl). Amplification conditions were as detailed above, with an annealing temperature of 56°C. Results were scored simply as HIV-1 proviral positive or negative, and statistical analyses were performed through the assignment of categorical variables as detailed above.

### RNA

#### Total RNA yield

The total RNA yield of the extracts (ng/µl) was assayed following initial DNAse treatment (as detailed below) using the RNA 6000 Nano LabChip kit with an Agilent 2100 Bioanalyzer (Agilent Technologies). Statistical analyses were performed on the absolute difference between RNA yields between paired specimens.

#### Amplifiable RNA quality via B2M qPCR assay

The total RT-PCR amplifiable RNA was measured through qPCR on cDNA reverse transcribed from the DNA/RNA extracts (see below for RT-PCR details) using primers B2MF (5′ TGACTTTGTCACAGCCCAAGATA) B2M R (5′ AATCCAAATGCGGCATCTTC) resulting in a 85bp amplification product. The B2M assay targets a commonly expressed house keeping gene and is specific for cDNA because it spans an intron. Details of the assay can be found in the RTPrimer database (ID: 152) [41]. SYBR green based qPCR reactions were undertaken as detailed above. As with the DNA qPCR assays, statistical analysis was performed on the difference in Ct values between the paired specimens.

#### Recovery of amplifiable HIV-1 RNA

The presence or absence of PCR amplifiable HIV-1 RNA was detected using the HIV-1 group M primers as detailed above, following an initial RT-PCR reaction with HIV-1 specific primers (see below for details). Results were scored and statistically tested as for the HIV-1 proviral assay.

#### RT-PCR reactions

Reverse transcription PCR was performed simultaneously for human B2M and HIV-1 *gag* fragments using the SuperScript III system (Invitrogen). Prior to reverse transcription 10 µl DNA extract was DNAse treated using DNAse I (Invitrogen) for 15 minutes at room temperature following the manufacturer's guidelines. 9 µl of this treated extract was added to 0.1 mM mixed dNTPs and 0.5 µl of primers G2 and B2MR (10 mM stock), mixed and incubated at 65°C for 5 minutes. The mixture was cooled on ice for 2 minutes, after which the following were added: 2.5 µl molecular biology grade H_2_O, 5 µl 5×RT-buffer, 1 µl Superscript III, 1 µl DTT. The mixture was incubated at 50°C for 90 minutes, then 55°C for 90 minutes. Subsequent PCRs and qPCRs were performed as detailed above, although with the incorporation of 2 µl of the cDNA to account for the 2-fold dilution of the original extract during cDNA synthesis.

## Results and Discussion

A summary of the results of the experiments, and p-values from statistical tests on the results are shown in supplemental table S1. The full results are extensive and can be supplied to the reader by request to the authors. At this point, we highlight that all the p-values reported in supplemental table S1 are based on 2-tailed tests, since many of the initial tests were performed in the absence of a hypothesis as to the direction of change that a method might confer. In other words, those p-values reflect whether there is any statistically significant difference in DNA/RNA quality between treatment and control pairs. Where appropriate in the discussion below, however, we report the p-values for significant results as for 1-tailed tests (the values are simply half those of the 2-tail tests); these p-values reflect significant post-manipulation improvements in DNA/RNA quality.

Furthermore, the table contains p-values of both parametric paired t-tests, and nonparametric Wilcoxon signed-ranked tests. The Wilcoxon signed-ranked tests broadly support the results of the t-tests (see supplemental table S1); we refer only to the t-tests in the discussion below. We also highlight that during the discussion we treat p-values of <0.05 as significant, but injecting some logic into our interpretations of statistical results based on the certainty that several relatively high p-values are stronger evidence against a null hypothesis than one moderately low value [42]. As our intention is to provide preliminary data that can be tested further by future studies, or which confirm the original reports describing the methods, we do not feel this is a serious challenge to our findings. Lastly, we note that due to the nature of our experimental design, the possibility exists that the different methods may have differential effects when applied with specific other methods. Although we have not investigated that formally here (due to lack of sample numbers to draw statistically supported conclusions), where observed in our data we comment on it.

### Authenticity of the data

It is widely appreciated that genetic analyses on samples that contain degraded DNA in general, and human DNA in particular, are faced with the considerable challenge of contamination with exogenous sources of DNA (e.g. [43], [44]). We believe that the results of this study are not compromised by contamination for a number of reasons. (i) The work was performed in a laboratory dedicated to working with degraded samples, under strict controls for contamination, including numerous extraction and PCR blanks that were always negative. (ii) More importantly however, we were able to investigate reproducibility among the results in several ways. Firstly, where used, the multiplex PCR/SNP typing produced genotypes for the tested samples, and these were always consistent within samples (data not shown). Secondly, the qPCR data was consistent within dilution series for individual DNA extracts. Thirdly, we observed consistent patterns of DNA survival between assays, for example when comparing mtDNA amplification success with nuDNA amplification success and proviral amplification success.

### The effect of deparaffinisation

Nucleic acid quality was tested on between 8 and 12 paired specimens in 8 different assays. Statistical analyses of the data indicate that there is no evidence that deparaffinisation of tissues confers any beneficial effect with regard to total yields of DNA and RNA extracted, amplifiable yield of mtDNA, nuDNA and RNA, maximum size of PCR amplifiable nuDNA fragment, or ability to PCR amplify proviral DNA or viral RNA. Therefore, we find no evidence that deparaffinisation using conventional 100% xylene (or alternative 100% pentane) washes is required for genetic applications that relate to any of the tested assays. This is in contrast to at least one previous finding [20] that reports PCR inhibition if paraffin is not removed. One simple explanation for the discrepancy is the age of that study; subsequent advances in nucleic acid extraction and amplification techniques may well have rendered this problem irrelevant. Hence, we recommend no deparaffinisation step be used.

### The effect of LiCO_3_ washing of Bouin's-fixed tissues

Nucleic acid quality was tested on 4 pairs of extractions using 5 different assays. Although this number may be too low to give definitive proof of effect, there is no statistical support of any benefit with regard to amplifiable yield of nuDNA or RNA, or maximum size of PCR amplifiable nuDNA fragment. These findings are in implicit agreement with other published studies that do not use LiCO_3_, yet report successful nucleotide recovery (e.g. [14]). However, although not confirmed with a significant p-value, the data provide a suggestion that the incorporation of a 27 mM LiCO_3_ wash results in a decreased total quantity of extracted DNA (47–58% decrease) and RNA (36–60% decrease) (1-tail paired t-tests p = 0.11 and 0.09 respectively). The expansion of this investigation on an increased number of specimens may resolve whether a significant effect exists. With our preliminary findings, however, we suggest that pre-washing of Bouin's-fixed samples with 27 mM LiCO_3_ confers no significant benefit for genetic applications that relate to any of the tested assays, and thus can be avoided.

### The effect of incubation at 98°C prior to enzymatic digestion

Nucleic acid quality was tested on between 8 and 24 paired specimens using 10 different assays. The data indicate that although preincubation as recommended by Wu et al. [21] does not significantly increases the PCR amplifiable RNA as assayed using qPCR, it does increase the ability to amplify cDNA derived from the HIV-1 RNA from the *gag* gene using primers HIVG1 and HIVG2 (1-tail paired t-test p<0.01). Presumably this difference is in some way linked to the differences between the cDNA synthesis and qPCR processes. Furthermore, the data provide additional evidence that preincubation might increase the yield of amplifiable nuclear DNA (1-tail paired t-test p = 0.05); however, we found that this treatment decreases the efficiency of the multiplex PCR assay (decrease in 1 to 3 SNPs, 1-tail paired t-test p = 0.04). These latter results seem both to confirm, and to conflict with observations of the study proposing this method [21]. In that study the authors reported a qPCR measurable increase in nuDNA yields post-treatment, and an increase in multiplex PCR efficiency. It is perhaps possible that this discrepancy is caused by the much larger size of our multiplex assay (44 amplicons in contrast to the 10 of the original study), plus differences in the way the multiplex results were assessed (gel visualization of PCR products in the original, but capillary electrophoresis SNP typing in this study).

### The effect of adding glycine to the digestion buffer

Nucleic acid quality was tested on between 8 and 12 pairs of samples using 8 different assays. Surprisingly, the data indicate that the PCR amplifiable nuDNA yields are significantly worse when 25 mM glycine is added to the buffer (up to 3 Ct values ≈8 times more DNA under a simplistic model of 100% PCR efficiency, 1-tail paired t-test p<0.01, average/standard deviation 1.4/1.3 Ct values). This trend was similarly observed in the mtDNA qPCR data (up to 4 Ct values ≈16 times more DNA under the same simplistic model, 1-tail paired t-test p = 0.03, average/standard deviation 1.2/1.6 Ct values). In the original study that describes glycine as a formaldehyde-binding agent [31], the authors report that glycine addition leads to moderately increased yield of DNA (although no details of quantity, or how this was measured are provided). Under the assumption that the measurement technique used measured total DNA in the extract, our data disagrees with this statement (1-tail paired t-test p = 0.20).

### The effect of incubation time on nucleic acid quality

**Figure 1:**
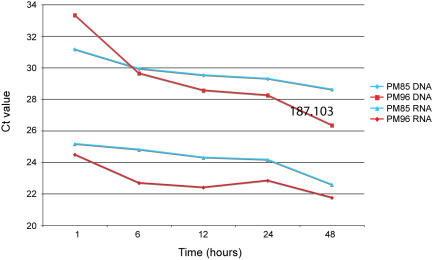
The association of levels of PCR amplifiable human nuDNA and RNA with digestion time. Note that in conventional quantitative real-time PCR assays, the measure of PCR amplifiable DNA or RNA (the Ct value) is inversely related to the starting concentration of template. Therefore, lower Ct values indicate higher original PCR amplifiable DNA or RNA yields.

Although we investigated the effect of digestion time on nucleic acid quality over a period of between 1 and 96 hours, due to experimental and practical limitations, the majority of our data comes from comparisons between 24 and 48 hour digests (8 measures of quality over 7–14 paired samples). Statistical analysis of this data strongly supports the finding that PCR amplifiable nuDNA and human RNA yields significantly increase as a result of increased digestion time (nuDNA over 3 Ct values, ≈8 times more DNA, 1-tail paired t-test p = 0.02; RNA over 2 Ct values, ≈4 times more RNA, 1-tail paired t-test p = 0.02). Although limited data do not allow statistical testing over the other time periods examined (1–96 hours), the data clearly show that the individual measurements from additional time points within these 48 hours agree with this finding (Figure 1). The finding that PCR amplifiable yields increase is logical and in agreement with the known fact that formaldehyde-protein cross-linking is reversible with the input of thermal energy into the system. Clearly, at a set temperature, increased time of incubation represents increased thermal energy input, thus greater cross-link reversal. Moreover, we found that total DNA or RNA yields were not affected by temperature. In other words, thermal energy does not lead to an increase of extracted nucleic acids; it merely makes whatever available DNA and RNA more amenable to amplification, presumably by making it less cross-linked.

We also find that in some samples the PCR amplifiable yields continue to improve as the digestion time is increased above 48 hours; however, this is variable, and in some cases the amplifiable levels of nucleic acids even appear to decrease at extended incubation. This may simply be a result of stochasticity, with the true underlying behavior representing a plateau effect. We speculate that this plateau will represent the level where all nucleic acids have been uncross-linked, and as such the limit to PCR becomes dependent on the specific the fragmentation that the nucleic acids have undergone during their history. With regard to practical implications of this finding, we suggest that incubations up to 48 hours or longer may well be useful. We do caution that, as thermal energy is an important factor with regard to nucleic acid degradation (e.g. [34]), this incubation should not be taken to excess. Our data (on a limited number of samples) shows no indication that even 96 hour digestions at 55°C adversely effect the DNA or RNA yields (both PCR amplifiable and total, data not shown). With increased incubation temperatures, however, (as observed, for example, in the next section) this may well become an important factor.

### The effect of digestion temperature on nucleic acid quality

Paired comparisons were made of the nucleic acid quality between digests performed at various temperatures between 55°C and 85°C. Initial comparisons contrasted 55°C and 65°C digests (8 comparisons, 4 to 8 pairs), and indicate that there is significant evidence that 65°C digests lead to increased levels of PCR amplifiable RNA (in the extreme up to 8 Ct values, ≈256 times more RNA, 1-tail paired t-test p = 0.01, average/standard deviation −5.3/8.7 Ct values). To some extent this is also reflected in the viral DNA results, with the success for amplification of HIV-1 *gag* gene correlating with the higher temperature digest (1-tail paired t-test p = 0.04). This finding is partially consistent with the findings on digestion time, indicating that the increased thermal energy leads to increased cross-link reversal. However, it remains unexplained why no significant effect was observed on the PCR amplifiable nuDNA yields. Of further interest, comparisons between digestions at 65°C and higher temperatures (75°C and 85°C) provide evidence that at the higher temperatures significant DNA and RNA degradation occurs (85°C versus 65°C, up to 8000 times less PCR amplifiable RNA, 1-tail paired t-test p<0.01, average/standard deviation −9.7/3.6 Ct values; up to 89% total RNA yield loss, 1-tail paired t-test p<0.01; up to 81% total DNA yield loss, 1-tail paired t-test p<0.01). Therefore, there is good evidence to argue that increased digestion temperature can be useful with regard to obtaining greater levels of PCR amplifiable RNA; however, these digestion temperatures should be limited to 65°C).

### Effect of nucleic acid extraction technique

Paired comparisons of nucleic acid quality were performed on extractions from a silica-column based DNA extraction kit (QIAamp DNA micro kit, Qiagen) and a conventional Tris-buffered proteinase k method followed by organic purification (8 comparisons, 8–18 paired samples). Interestingly, although the silica-based extraction kit used was a dedicated DNA extraction kit, we find strong evidence not only that it is effective for RNA extraction, but that its use leads to increased levels of PCR amplifiable human RNA compared with organic extraction (up to 4 Ct values, ≈16 times more template, 1-tail paired t-test p<0.01, average/standard deviation −2.0/1.6 Ct values). Furthermore, the data also provide three sources of evidence (with at least marginal statistical support) that, compared to the organic alternative, the extractions performed using this kit contain higher levels of PCR amplifiable nuDNA (qPCR assayed nuDNA 1-tail paired t-test p = 0.08; qualitative assay of 106/112 bp Amelogenin fragment 1-tail paired t-test p = 0.04; Multiplex PCR efficiency 1-tail paired t-test p = 0.05). Interestingly, similar increases in efficiency of silica based extraction methods have also been previously reported in studies on ancient bone [45]. The observation of this study, in combination with observations of no apparent effect on the total levels of extracted DNA and RNA therefore suggest that the components of the QIAamp extraction buffers have enhanced nucleic acid-protein cross-link reversal properties compared to the conventional buffers. The apparent lack of effect on longer DNA PCR products, however, indicates that in the specimens examined here, the effect of DNA fragmentation again places an upper limit on the improvements that can be made.

That RNA is co-extracted using the Qiagen kit may seem surprising (given the kit is marketed by the vendor as a DNA extraction kit, and that considerably more expensive commercially available dedicated RNA extraction kits also exist). However, under the conditions of the kit, the silica column will bind to both DNA and RNA. Thus, in the absence of RNAses, there is no reason why the RNA would be degraded to any greater extent than DNA. Thus, it would seem that the manufacturing of the QIAamp kit is under conditions that are suitably controlled to exclude RNAses.

### Effect of Taq based DNA repair

The effect of using *Taq* polymerase to increase the quality of the extracted DNA [14], [35] was investigated on 31 samples using qPCR of nuDNA, and 42 pairs of extracts using conventional PCR assays for 106/112 bp and 212/218 bp fragments of Amelogenin. Although the results do not provide significant support for increasing the size of amplifiable DNA (106/112 bp fragment, 1-tail paired t-test p = 0.16, 212/218 bp fragment no observable difference), a significant increase in the levels of PCR amplifiable nuDNA was observed (up to 2 Ct values, ≈4 times more template DNA, 1-tail paired t-test p<0.01, average/standard deviation −0.9/1.3 Ct values). This observation was consistent across all types of extraction tested, including both formalin fixed and Bouin's-fixed specimens, clearly supporting the published reports as to its efficacy. We note, however, that in the previous publications no qPCR was used; the efficacy was simply evaluated qualitatively through increased PCR success. The inability to improve on size of PCR amplicon in our study therefore provides strong evidence that a major factor limiting success in the samples investigated here is DNA fragmentation within the specimens, a factor that naturally will vary between fixed specimens investigated. In other words, although minor damage to extracted DNA can evidently be repaired, more serious degradation, such as fragmentation, might impose unavoidable limitations on the maximum length of PCR amplicons. Furthermore, we raise here the additional observation that both our and the previous data [14], [35], do not provide any information as to potential sequence errors introduced during the repair process, for example due to misincorporation of nucleotides opposite common forms of DNA damage such as hydrolytically damaged cytosine nucleotides [46], [47]. To investigate this further would involve extensive cloning and resequencing of the repaired amplicons, which is beyond the scope of this study, although comparison of the qPCR dissociation curves from extracts both before and after repair hints at the fact that no observable sequence differences exist. However, this observation may simply be an artefact of the relatively large levels of template molecules that we have in our extracts, in comparison to those found in other studies of degraded materials, and thus we recommend that future studies directly address this issue.

### Critical point dehydration and hot-alkali incubation

Due to limited samples and facilities, insufficient analyses were performed to enable statistical testing of the results from the critical point dehydration (28) and hot-alkali treatments [22], [23]. However, from our investigation on a limited number of samples we found no proof that critical point dehydration confers any effect on the quality of the DNA (measures included total DNA and RNA yield, qPCR amplifiable B2M RNA, size of PCR amplicon and HIV proviral assays). This finding is in stark contrast to the incredible results reported in the original study-a full ethanol dehydration series followed by critical point dehydration on samples aged between 16 and 70 years led to the recovery of DNA fragments up to 194 kb in length, and the generation of nuDNA PCR fragments of nearly 2,000 bp from samples which previously yielded no amplicons. In light of the fact that no apparent plausible explanation exists as to why critical point dehydration should convey these unprecedented benefits, and our spectacular lack of any such improvements in any of the DNA quality assays, we question whether the results of the initial study [28] are valid.

**Figure 2:**
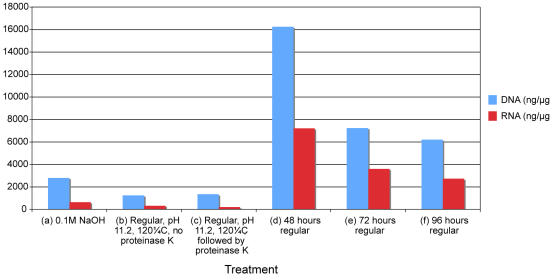
DNA and RNA yields (ng/µg original tissue) in six different nucleic acid extracts from sample PM85L. Each extract derives from a different digestion technique (a–f). For full details of the digestion protocols refer to main text. The data clearly demonstrates that total (but not necessarily amplifiable) DNA and RNA yields are greatly reduced in extracts performed using hot alkali techniques [22], [23].

**Figure 3:**
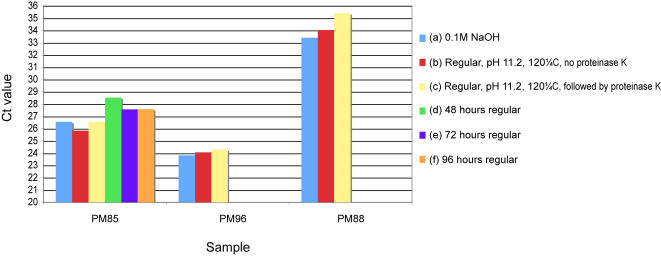
The relative PCR-amplifiable nuDNA yields resulting from different extraction techniques (a–f) on 3 different formalin fixed samples. Only sample PM85 was extracted with techniques (d–f). Techniques (a–c) are based on the hot-alkali methods of Shi et al. [22], [23], while techniques (d–f) are conventional proteinase k digestion techniques with extended digestion times. Note that in conventional quantitative real-time PCR assays, the measure of PCR amplifiable DNA (the Ct value) is inversely related to the starting concentration of DNA. Therefore, lower Ct values indicate higher original PCR amplifiable DNA yields. Although too small a sample to provide statistically supported findings, the data suggest that while there is little difference between the efficacies of the different hot alkali techniques themselves, they provide marginally higher levels of PCR amplifiable nuDNA.

On the other hand, with regard to the hot-alkali treatments advocated by Shi et al. [22], [23], our data is generally in agreement with the original published reports. As in the original publications [22], [23] we find that incubation at increased temperature and pH (120°C, buffer adjusted to pH 11.2 or 0.1 M NaOH solution) gives the highest levels of PCR-amplifiable nuDNA, although at a cost to the total DNA extracted (Figure 2, Figure 3). Furthermore, we find that in comparison to our own variants of the method (additional proteinase k digestion post alkali/heat treatment) or extended digestion time (48–96 hours) the Shi et al. [22], [23] methods also result in marginally increased yields of PCR amplifiable nuDNA (Figure 3).

However, our data also indicate that this success comes at a serious cost–predictably our results reveal that the application of heat and/or alkali in this way rapidly degrades the total RNA within the sample to sub-detectable levels (Figure 2). In light of the instability of DNA and RNA in both hot and alkali conditions, this is not surprising, but it does suggest that a trade-off is occurring in the extracted DNA between cross-link reversal and DNA degradation, and that a careful balance is required to ensure that PCR amplifiable DNA remains after treatment.

### Conclusions

The aim of our study was to provide direct comparisons of a large number of methods that have been proposed for use on fixed tissues. While the findings are to some degree limited by sample size and by the nested approach adopted, they demonstrate that the extraction methods used in genetic studies of fixed tissues need to be chosen carefully, in light of the specific endpoints of particular investigations. Briefly, several labor- and resource-intensive techniques with supposed benefits for formalin- or Bouin's-fixed specimens appear to be, at best, a waste of time and energy. These include deparaffinisation, LiCO_3_ washing, and critical point drying. Some methods, like the use of glycine in digestion buffers, may even seriously decrease amplifiable DNA. Still other techniques do have significant benefits, often increasing the effective level of template DNA/RNA by many fold. These include pre-extraction incubation at high temperature; optimal digestion temperature; long (48 h) duration of digestion; silica-based extraction; DNA repair using *Taq* polymerase; and hot-alkali extraction. Importantly, though, many of these benefits have trade-offs. Increased digestion time, temperature, and pH all appear to liberate amplifiable nucleic acids, most likely by reversing formalin-induced cross-links; however, these same conditions appear to deplete the total pool of nucleic acids available for amplification. While we encourage further investigations on larger datasets to confirm and extend our findings, for the convenience of future studies on fixed materials Table 2 summarizes our findings in a manner that we hope will assist with the choice of methods.

**Table 2:** Summary of positive (+) and negative (-) effects of tested methods on different measures of nucleic acid quality.

	Paraffin removal	LiCO_3_ wash	98°C pre incubation	Glycine	Increased incubation time	65°C digestion temperature	>65°C digestion temperature	QIAamp	Taq repair	Critical point drying	Hot alkali*
**Total DNA**		-					---				---
**Amplfiable DNA**		not tested	++	---	+++			+	+++		+
**Increased DNA amplicon size**								++			+
**Multiplex PCR**	not tested	not tested	--	not tested				++	not tested	not tested	
**Proviral DNA**		not tested				++			not tested		
**Total RNA**	not tested	-		not tested			---		not tested		
**Amplifiable RNA**					++	+++	---	+++	not tested		---
**Viral RNA cDNA**		not tested	+++		not tested	not tested	not tested	not tested	not tested	not tested	not tested

The statistical support behind the observations is for the most part indicated as follows. +/- Effect suggested by data, ++/-- Statistical support of observation if no Bonferroni correction applied; +++/--- Strong statistical support of observation. *Observations for the hot-alkali treatment are based solely on the raw data and have not been statistically tested.

## Supporting Information

Table S1 Summary of test results. The table indicates the number of compared comparisons, and the p-values of both the parametric paired 2-tail t-tests and the non parametric Wilcoxon signed-ranked tests.
(0.03 MB XLS)

Table S2 (0.08 MB XLS)
